# Parental management of childhood fever and associated factors in southeastern Brazil: a cross-sectional study

**DOI:** 10.1016/j.jped.2026.101581

**Published:** 2026-07-28

**Authors:** Lucas Abreu Dias, Guilherme Henrique Silva Oliveira, Alisson Rafael de Oliveira Pereira, Luiz Otávio de Oliveira Pala, Tathiana Tavares Menezes, Luciano José Pereira

**Affiliations:** a Universidade Federal de Lavras, Lavras, MG, Brazil; b Universidade Federal de Lavras, Department of Medicine, Lavras, MG, Brazil; c Universidade Federal de Lavras, Department of Statistics, Lavras, MG, Brazil

**Keywords:** Fever, Antipyretics, Caregivers, Surveys and questionnaires, Health knowledge, Attitudes, Practice, Pediatrics

## Abstract

**Objective:**

To evaluate parental and caregiver practices in the management of childhood fever and to identify factors independently associated with appropriate fever management according to Brazilian Pediatric Society recommendations.

**Methods:**

A cross-sectional study was conducted in 2024 using a structured questionnaire administered both online and in person at primary health care units. A total of 847 parents or caregivers of children and adolescents participated in the study. Fever management was considered appropriate only when all predefined diagnostic and therapeutic criteria were met simultaneously. Associations were assessed using chi-square tests and multivariable logistic regression.

**Results:**

60.7% (n = 514) of responses were collected online and 39.3% (n = 333) in person. Only 42% of caregivers fulfilled all predefined criteria for appropriate fever management. In bivariate analyses, appropriate management was associated with both educational level and time elapsed before seeking medical care. However, after multivariable adjustment, educational level was no longer significantly associated with the outcome, whereas only time to medical consultation remained independently associated. Compared with immediate consultation, caregivers who waited 6–12 h (OR = 1.91; 95% CI: 1.16–3.16), 12–24 h (OR = 1.96; 95% CI: 1.20–3.20), or 24–36 h (OR = 1.67; 95% CI: 1.02–2.75) were more likely to manage fever appropriately.

**Conclusions:**

Less than half of caregivers managed childhood fever appropriately. A short period of observation before seeking medical care was independently associated with better adherence to recommended practices. These findings highlight persistent gaps in home-based fever management and suggest that caregiver decision-making regarding healthcare-seeking may be a relevant target for future educational interventions.

## Introduction

Fever is one of the most common clinical manifestations in pediatric care, accounting for approximately 20–30% of consultations in both outpatient and emergency settings [1]. Despite its high prevalence, significant gaps persist in caregiver knowledge regarding the appropriate management of febrile episodes in childhood.

This limited understanding contributes to increased anxiety among parents and caregivers, as fever is frequently perceived as a sign of potentially severe conditions, such as febrile seizures, fever without a source, and infectious diseases [1]. Such perceptions favor the development of the phenomenon known as *fever phobia*, characterized by disproportionate and irrational fear related to fever itself rather than its underlying cause [2]. In addition, inappropriate management of fever in children has been associated with several adverse outcomes, including misuse of antipyretic medications, dosing errors, increased risk of adverse drug events and interactions, and unnecessary medical consultations. Collectively, these factors contribute to the overuse of health services and increased healthcare costs [3,4].

Although evidence-based clinical guidelines establish safe and effective parameters for the management of fever in the pediatric population [5], recent studies conducted in both Brazilian and international settings continue to demonstrate persistent misconceptions and inadequate caregiver practices regarding childhood fever management, including inappropriate antipyretic use, excessive concern regarding fever-related complications, and inconsistent healthcare-seeking behaviors [[6], [7], [8], [9]]. Contemporary evidence also suggests that educational, informational, and sociocultural factors may influence caregiver decision-making and home-based fever management practices [[9], [10], [11]]. A systematic review encompassing 36 studies and over 26,000 participants across four decades found that non-evidence-based fever management practices have remained relatively stable despite educational interventions, and identified physicians as the predominant source of information sought by caregivers, while friends, family, and personal experience were also among the most frequently reported sources — patterns that may perpetuate outdated or inaccurate practices [11]. Internet use has grown substantially as an additional resource in recent years, raising further concerns about the reliability of content accessed by caregivers [11].

In this context, health education strategies are considered essential to promote rational use of pharmacological therapies and to reduce avoidable demand for medical care [8,9]. The assessment of family perceptions and practices related to childhood fever plays a strategic role in public health planning, serving as a diagnostic tool to identify weaknesses in home-based fever management and to support the development of educational interventions tailored to the local sociocultural context [10].

Based on previous evidence, caregiver sociodemographic characteristics - particularly educational level - and healthcare-seeking behaviors may be associated with home-based fever management practices. More specifically, it is expected that higher educational attainment and less immediate healthcare-seeking behavior would be associated with greater adherence to recommended fever management practices. However, the mechanisms underlying these associations remain incompletely understood, particularly in regional Brazilian contexts [11]. Therefore, the present study aimed to analyze the practices adopted by parents and caregivers in response to pediatric fever and to identify factors associated with appropriate fever management, as well as potential targets for future educational interventions.

## Methods

This was a cross-sectional survey study conducted between January and July 2024, evaluating therapeutic practices adopted by parents or caregivers in the management of febrile episodes in children and adolescents. The manuscript was prepared in accordance with the STROBE guidelines [12], as presented in Supplementary Table 1. The study was approved by the Research Ethics Committee of the Federal University of Lavras (CAAE 74611323.0.0000.5148) and conducted in compliance with Brazilian National Health Council Resolution 466/12.

The study was carried out in Lavras, a medium-sized municipality in southern Minas Gerais, Brazil, approximately 240 km from Belo Horizonte, with an estimated population of 110,682 inhabitants, with high schooling rates and a favorable level of social development.

Data were collected using a structured questionnaire adapted from the instrument proposed by Gomide-Nogueira de Sá [10,13], originally based on the fever-management tool developed by Walsh, Edwards, and Fraser in Australia [14]. The assessment of parental fever-management practices has also been supported by the development and validation of dedicated measurement instruments in other populations [15]. The questionnaire also collected information regarding child age, congenital conditions, comorbidities, and previous medical history, variables potentially related to caregiver fever-management behaviors. The original Australian instrument underwent a formal methodological development process, including literature review, incorporation of previously published fever-management tools, semi-structured discussions with parents, expert assessment of content and face validity, test-retest reliability analysis, and exploratory factor analysis of the Parent Fever Management Scale (PFMS) [14].

The Brazilian adaptation underwent pilot testing and iterative refinement before application in Brazilian caregivers [10,13]. The present study performed a contextual adaptation to reflect contemporary fever-management practices, current thermometer technologies, and the epidemiological characteristics of the target population, without a new formal psychometric validation process. All questionnaire modifications introduced in the present study are detailed in the supplementary material and are available as Supplementary Appendices 1 and 2.

Sample size was calculated assuming an unknown population size, a margin of error of 5%, a 95% confidence level, and an estimated thermometer use prevalence of 76.7% [13], yielding a minimum required sample of 278 participants. A non-probabilistic convenience sampling strategy was adopted, with broad community dissemination across social and healthcare settings to increase sample heterogeneity; each participant was included only once.

The questionnaire comprised 38 items covering sociodemographic characteristics, medical history, fever management practices, beliefs, risk perceptions, and information sources. Data was collected through online and in-person administration of the same questionnaire. The online version was made available via the Google Forms® platform, while the printed version was administered to users attending Primary Health Care units of the Brazilian Unified Health System.

For online participants, the full Informed Consent Form was displayed as the first section of the questionnaire; participants were required to explicitly confirm their agreement before accessing any study items. For in-person participants, a printed version of the same form was provided and signed prior to questionnaire completion. Both procedures were conducted in accordance with Brazilian National Health Council Resolution 466/2012, which recognizes electronic informed consent as a valid modality for digitally administered research. The project was approved by the Human Research Ethics Committee of UFLA under CAAE number 74611323.0.0000.5148.

**Figure 1:**
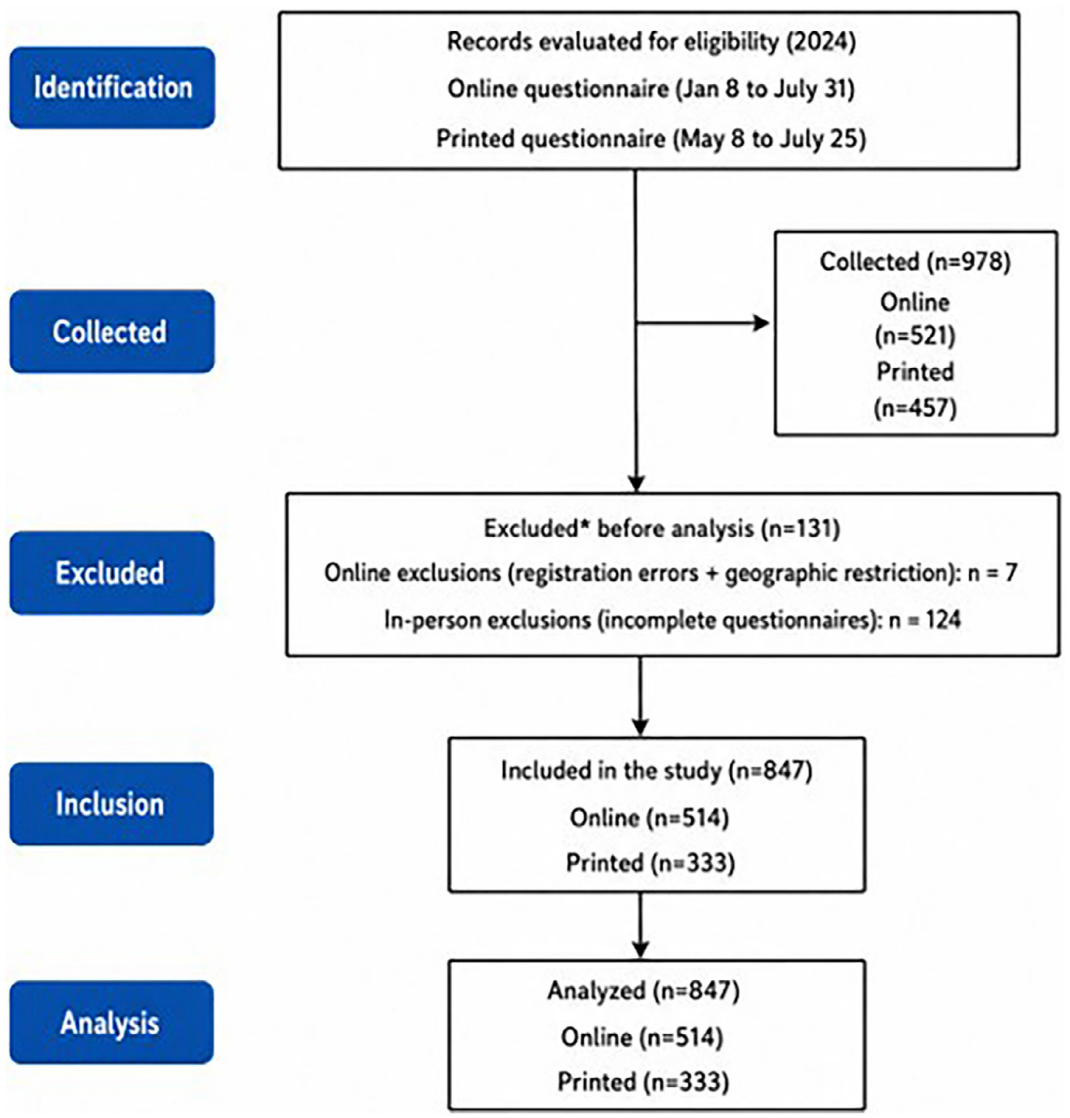
Flow diagram of participant recruitment, exclusions, and inclusion in the final analysis according to STROBE recommendations. * Geographic restriction, mandatory response settings (including exclusion of questionnaires with even a single missing response), and prior fever management experience were applied as automatic eligibility filters in the online questionnaire (Google Forms®). In the in-person component, incomplete responses resulted from participants being called for their medical appointment before completing the questionnaire. Primary Health Care units operate under the territoriality principle of the Brazilian Unified Health System (SUS), inherently restricting the in-person sample to Lavras residents. The number of exclusions per criterion was not individually recorded, as the filtering mechanisms were applied automatically and simultaneously during data collection.

Eligible participants were parents or caregivers of children aged 0–18 years residing in Lavras, who had managed at least one febrile episode. Of 978 questionnaires initially collected, 847 were included after exclusion of incomplete, duplicate, inconsistent, or ineligible responses (Fig. 1).

The primary outcome was defined as appropriate management of a pediatric febrile episode, including both appropriate fever diagnosis and appropriate treatment, according to the most recent recommendations of the Brazilian Pediatric Society [1]. Appropriate diagnosis comprised five criteria: possession of a thermometer; use of at least a digital thermometer; correct knowledge of its use; axillary temperature measurement; and definition of fever as a temperature ≥ 37.5 °C. Appropriate treatment comprised four criteria: use of a recommended antipyretic; exclusive use of dipyrone, paracetamol, or ibuprofen without combination; dose calculation based on current body weight; and non-use of antibiotics. Fever management was classified as appropriate only when all nine criteria were simultaneously fulfilled. This dichotomous composite structure was adopted to provide a standardized and reproducible operational definition suitable for inferential statistical analysis in a large cross-sectional dataset. The questionnaire did not include indications for antipyretic use in cases of fever associated with malaise or prostration, as recommended by the Brazilian Pediatric Society [1], and this variable was therefore excluded from the outcome definition.

Variables included in presentation and association analyses were selected based on clinical plausibility, epidemiological relevance, analytical feasibility, and potential association with the outcome. Behavioral variables analyzed included mode of data collection, regular medication use, history of febrile seizure, time elapsed before seeking medical care, information source guiding medication decisions, use of non-pharmacological methods, fever perception, and general information source. Sociodemographic variables included profession, race, relationship to the child, exclusive public health system use, number of children under care, housing status, income, and educational level.

Statistical analyses were performed using R software, version 4.5.1 (2025) [16], with the dplyr [17] and epiDisplay [18] packages. Chi-square tests assessed associations between each variable and the outcome; variables with a p < 0.25 were selected as candidates for multivariable logistic regression [19]. This threshold was adopted to avoid premature exclusion of potentially relevant confounding variables [19].

Candidate variables included mode of data collection, time elapsed before seeking medical care, source of information guiding medication decisions, perception of fever as beneficial, sources used to seek information about fever, relationship to the child, exclusive public health system use, housing status, and educational level. Variable selection was subsequently performed using the Akaike Information Criterion (AIC) through a stepwise algorithm [20] to balance model parsimony and explanatory adequacy. The final model retained only the time elapsed before seeking medical care as independently associated with appropriate fever management.

## Results

A total of 847 parents or caregivers participated in the study, of whom 514 (60.7%) completed the questionnaire online and 333 (39.3%) in person. The overall sociodemographic and clinical profile of the study sample is presented in Table 1. Overall, appropriate fever management was identified in 356 participants, corresponding to 42% of the sample.

**Table 1:** Sociodemographic characteristics of the study participants.

Variable	n (%) or mean (SD) or median (IQR)
**Caregiver characteristics**	
Age, years	median 36 (IQR 30–41); mean 36.07 (SD 8.32)
**Self-reported race**	
Asian	24 (2.83)
White	409 (48.29)
Indigenous	3 (0.35)
Brown/Mixed-race	295 (34.83)
Black	116 (13.70)
**Relationship to child**	
Mother	702 (82.88)
Father	91 (10.74)
Other relative	21 (2.48)
Legal guardian	33 (3.90)
**Marital status**	
Married/Common-law marriage/Cohabiting	575 (67.88)
Separated/Divorced	57 (6.73)
Single	177 (20.90)
Widowed	6 (0.71)
Other	32 (3.78)
**Educational level**	
No formal education	1 (0.12)
Elementary school	76 (8.97)
High school	366 (43.21)
Higher education	211 (24.91)
Postgraduate education	193 (22.79)
**Profession**	
Healthcare professional	102 (12.04)
Non-healthcare professional	745 (87.96)
**Number of children**	
0	14 (1.65)
1	373 (44.04)
2	319 (37.66)
3	109 (12.87)
4 or more	32 (3.78)
**Number of people in the household**	
One or two	84 (9.92)
Three or four	599 (70.72)
Five or six	143 (16.88)
More than six	21 (2.48)
**Child characteristics**	
Age of the eldest child, years	median 7 (IQR 3–12); mean 7.75 (SD 5.89)
**Sex of children under care**	
Female only	298 (35.18)
Male only	308 (36.36)
Both sexes	241 (28.45)
**Exclusive dependence on the public health system (SUS)**	
Yes	433 (51.12)
No	414 (48.88)
**Presence of comorbidities**	
Yes	106 (12.51)
No	741 (87.49)
**Presence of congenital conditions**	
Yes	29 (3.42)
No	818 (96.57)
**Regular medication use**	
Yes	202 (23.85)
No	645 (76.15)
**History of febrile seizures**	
Yes	54 (6.38)
No	793 (93.62)

Data are presented as n (%) or mean (SD). SD, standard deviation; SUS, Brazilian Unified Health System.

Regarding the individual components of the composite outcome, detailed response distributions are presented in Supplementary Table 2, while adherence and failure frequencies according to the predefined operational criteria are shown in Supplementary Table 3. The highest frequencies of failure were observed for inappropriate antipyretic selection/combination practices (31.4%) and inaccurate fever definition thresholds (30.1%).

In contrast, the highest adherence frequencies were observed for correct thermometer handling, having a thermometer at home, and absence of antibiotic use. Regarding comparison between data collection modalities, an initial chi-square analysis did not suggest an association between mode of data collection and fever management categories (p = 0.228), as shown in Table 1. Consistently, no statistically significant differences were observed between online and in-person respondents in the distribution of the primary outcome or key sociodemographic variables.

In the bivariate analysis, appropriate fever management was significantly associated with the time elapsed before seeking medical care (p = 0.025) and educational level (p = 0.010). No statistically significant associations were observed for the remaining variables evaluated (Table 2, Table 3). Variables with p-values < 0.25 were included in the multivariable logistic regression model.

**Table 2:** Bivariate analysis of factors associated with appropriate childhood fever management.

Variable	Category	Appropriate management		p-value
		Yes (%)	No (%)	
Data Collection	Google Forms®	225 (43.8)	289 (56.2)	**0.228**
	USF/UBS	131 (39.3)	202 (60.7)	
Regular use of medication/supplements	No	270 (41.9)	375 (58.1)	0.922
	Yes	86 (42.6)	116 (57.4)	
History of febrile seizure	No	337 (42.5)	456 (57.5)	0.362
	Yes	19 (35.2)	35 (64.8)	
Time to seek medical care (hours)	Immediate consultation	36 (31.6)	78 (68.4)	**0.025**
	6–12	76 (46.6)	87 (53.4)	
	12–24	86 (47.3)	96 (52.7)	
	24–36	75 (43.9)	96 (56.1)	
	36–48	27 (31.4)	59 (68.6)	
	48–72	56 (42.7)	75 (57.3)	
Source of information to decide medication	Family member	9 (33.3)	18 (66.7)	**0.099**
	Pharmacist	46 (38.0)	75 (62.0)	
	Physician	284 (44.4)	356 (55.6)	
	Media/information outlets	15 (31.2)	33 (68.8)	
	Other	2 (18.2)	9 (81.8)	
Non-pharmacological method	Bathing	295 (42.3)	402 (57.7)	0.357
	Water compress	20 (37.0)	34 (63.0)	
	Alcohol compress	17 (56.7)	13 (43.3)	
	None	21 (38.2)	34 (61.8)	
	Other	3 (27.3)	8 (72.7)	
Is fever beneficial?	No	117 (39.0)	183 (61.0)	**0.211**
	Yes	239 (43.7)	308 (56.3)	
Is fever dangerous?	No	59 (46.1)	69 (53.9)	0.361
	Yes	297 (41.3)	422 (58.7)	
Sources used to seek information about fever	Scientific articles	16 (42.1)	22 (57.9)	**0.175**
	Internet	291 (43.8)	373 (56.2)	
	Books	14 (32.6)	29 (67.4)	
	Other	35 (34.3)	67 (65.7)	

Values are presented as n (%).

**Table 3:** Multivariable logistic regression analysis of factors associated with appropriate childhood fever management.

Variable	Category	Appropriate management		p-value
		Yes (%)	No (%)	
**Occupation**	Health-related field	44 (43.10)	58 (56.90)	0.893
	Other	312 (41.90)	433 (58.10)	
**Race**	Asian	11 (45.80)	13 (54.20)	0.393
	White	183 (44.70)	226 (55.30)	
	Indigenous	2 (66.70)	1 (33.30)	
	Brown (Pardo)	117 (39.70)	178 (60.30)	
	Black	43 (37.10)	73 (62.90)	
**Relationship to child**	Mother	305 (43.40)	397 (56.60)	**0.185**
	Father	32 (35.20)	59 (64.80)	
	Legal guardian	19 (35.20)	35 (64.80)	
**Exclusive use of the public health system (SUS)**	No	183 (44.20)	231 (55.80)	**0.237**
	Yes	173 (40.00)	260 (60.00)	
**Number of children under care**	0	2 (28.60)	5 (71.40)	0.478
	1	158 (43.20)	208 (56.80)	
	2	145 (43.50)	188 (56.50)	
	3	41 (38.00)	67 (62.00)	
	≥ 4	10 (30.30)	23 (69.70)	
**Housing status**	Rented	120 (46.30)	139 (53.70)	**0.099**
	Provided (loaned)	30 (42.30)	41 (57.50)	
	Other	0 (0.00)	5 (100.00)	
	Owned/financed	206 (40.20)	306 (59.80)	
**Household income**	No fixed income	21 (35.60)	38 (64.40)	0.336
	1–2 minimum wages	101 (38.80)	159 (61.20)	
	2–5 minimum wages	150 (45.00)	183 (55.00)	
	> 5 minimum wages	84 (43.10)	111 (56.90)	
**Educational level**	None	0 (0.00)	1 (100.00)	**0.01**
	Primary education	22 (28.90)	54 (71.10)	
	Secondary education	146 (39.90)	220 (60.10)	
	Higher education	188 (46.50)	216 (53.50)	

After variable selection using the Akaike Information Criterion, only the time elapsed before seeking medical care remained independently associated with appropriate fever management (Table 4; Supplementary Figure 1). These findings indicate that an intermediate delay before seeking medical care was consistently associated with greater adherence to recommended fever management practices.

**Table 4:** Performance of the final multivariable logistic regression model.

Variable	Category	Estimate (SE)	OR (CI-95%)
**Time elapsed before seeking medical care** (reference = Immediate consultation)	6 – 12 h	0.648* (0.256)	1.91 (1.16 - 3.16)
	12 – 24 h	0.671** (0.251)	1.96 (1.20 - 3.20)
	24 - 36 h	0.513* (0.254)	1.67 (1.02 - 2.75)
	36 – 48 h	−0.021 ns (0.308)	0.98 (0.54 – 1.79)
	48 – 72 h	0.468 ns (0.080)	1.60 (0.94 – 2.70)

OR, odds ratio; CI, confidence interval; SE, standard error; ns, not significant.

* p < 0.05; ** p < 0.01.

Compared with immediate medical consultation, caregivers who waited 6–12 h after identifying fever had 1.91 times higher odds of appropriate home-based fever management (OR = 1.91; 95% CI: 1.16–3.16). Similarly, waiting 12–24 h was associated with a 1.96-fold increase in the likelihood of appropriate management (OR = 1.96; 95% CI: 1.20–3.20), while waiting 24–36 h was associated with a 1.67-fold higher likelihood (OR = 1.67; 95% CI: 1.02–2.75) (Supplementary Figure 1). Among caregivers who waited 48–72 h, a positive but non-statistically significant association was observed (OR = 1.60; 95% CI: 0.94–2.70), suggesting a possible trend that warrants cautious interpretation (Table 3). No significant differences in the odds of appropriate fever management were observed among caregivers who waited >36 h before seeking medical care (Table 3).

An additional complementary analysis comparing caregivers of children with and without reported chronic or congenital conditions was performed to further evaluate this potential confounding factor. Overall, 13.93% of respondents (n = 118) reported caring for children with at least one chronic or congenital condition. The most frequently reported conditions were asthma/bronchitis (n = 39), followed by autism spectrum disorder (n = 5) and epilepsy (n = 2). Other conditions, including heart disease, Hashimoto’s thyroiditis, and chronic lung disease, among others, were reported at low frequencies, with only one occurrence each. No statistically significant association was observed between fever management practices and the presence of comorbidities or congenital conditions (p = 0.935).

## Discussion

The present study evaluated parental and caregiver practices in the management of pediatric fever and identified a low proportion of fever management considered appropriate (42%), based on criteria derived from the most recent recommendations of the Brazilian Pediatric Society [1]. This finding highlights the persistence of inadequate practices related to both diagnosis and treatment, despite the wide availability of information and consolidated clinical guidelines [1,5,8].

With respect to diagnosis, failures in thermometer use, adoption of non-recommended measurement routes, and use of incorrect temperature cutoffs compromise accurate fever identification [13], favoring unnecessary or potentially harmful interventions. Inadequate recognition of fever, therefore, represents a critical element in the cascade of erroneous decisions related to home-based fever management, leading to consequent errors in antipyretic administration, regardless of educational level or access to health services [10,13]. These findings indicate that the availability of resources alone is insufficient to ensure appropriate fever management. These patterns are corroborated by a recent large-scale survey (n = 3133) by Cengiz & Gündüz [4], which identified widespread failures in thermometer use and fever definition among mothers, alongside high parental anxiety scores — with febrile seizures reported as the most feared complication, mirroring the concerns described in the present study.

Recently, the Brazilian Society of Pediatrics updated the definition of fever, considering axillary temperature ≥ 37.5 °C [1], corresponding to approximately 38.0 °C when measured orally or rectally [21]. Although this update represents an advance in diagnostic standardization, changes in cutoff values may initially generate uncertainty among caregivers and healthcare professionals, particularly given international variability in fever definitions and limited access to updated guidelines [21]. In this context, the definition of fever is not based on a single absolute value, but rather on an increase in body temperature resulting from elevation of the thermoregulatory set point, varying according to the measurement method, population, and current guidelines [1]. The importance of continuous professional updating and adequate dissemination of recommendations is therefore reinforced.

Regarding treatment, the persistence of non-recommended practices, including inappropriate use of antipyretics and physical methods lacking scientific support, reflects the continued influence of fever phobia [21]. Recent reviews indicate that commonly used interventions such as cold baths, compresses, and adhesive cooling patches remain widely adopted despite limited evidence of clinical benefit or even potential contraindications; [22] and may promote excessive interventions leading to irritability, shivering, and vasomotor changes in children [22,23]. The qualitative synthesis by Vicens-Blanes et al. [6] further contextualizes these findings, identifying that parental fear of fever is deeply rooted in cultural beliefs and prior negative experiences, and that such perceptions systematically override factual knowledge in home-based decision-making — a mechanism that may help explain the persistence of non-recommended practices regardless of educational attainment.

In the multivariable analysis, only the time elapsed before seeking medical care remained independently associated with appropriate fever management. Greater adequacy was observed among caregivers who waited an intermediate interval before seeking care, suggesting a behavioral pattern without allowing inference regarding caregiver competence or understanding of the fever course [22]. Immediate medical consultation, in contrast, may reflect anxiety, insecurity, or limited autonomy in home-based fever management [2,9,11,22]. Consistent with the present findings, Cengiz & Gündüz [4] also documented that anxiety regarding fever complications was a primary driver of early and frequent healthcare-seeking, independent of actual fever severity.

Although caregivers who waited 48–72 h before seeking medical care did not demonstrate a statistically significant association with appropriate fever management, the direction of the effect estimate remained consistent with that observed for intermediate waiting intervals. This finding may reflect limited statistical precision rather than a true absence of association. Alternatively, excessively prolonged delays may represent a distinct behavioral profile influenced by barriers to healthcare access, underestimation of symptom severity, or different perceptions regarding illness progression. Given the overlapping confidence intervals and the cross-sectional design, these interpretations should be considered exploratory and hypothesis-generating only.

In this context, the association between an intermediate waiting interval and appropriate fever management may be interpreted in light of the conceptual framework of safety-netting. Importantly, safety-netting was not an intervention component of the study nor directly measured, but rather used as a post hoc interpretive lens to contextualize caregiver decision-making under conditions of clinical uncertainty [24]. From this perspective, caregivers who adopt an intermediate waiting interval may demonstrate greater ability to monitor symptom evolution and distinguish self-limited illness from conditions requiring medical evaluation, potentially contributing to more appropriate healthcare utilization.

Educational level was marginally associated with appropriate fever management in bivariate analysis but did not remain significant in the multivariable model. Previous studies have demonstrated an association between health literacy and educational level, indicating that higher educational attainment tends to favor better comprehension and application of health-related guidance [25]. Corroborating this complexity, Menekşe et al. [8] found that parental health literacy was directly associated with fever management quality, yet highlighted that this relationship is mediated by contextual and behavioral factors, suggesting that literacy alone does not fully determine management adequacy, a finding consistent with the present results.

However, evidence also indicates that inappropriate fever management practices persist even among healthcare professionals [[25], [26], [27], [28]]. In a recent survey involving 620 Italian pediatricians, Chiappini et al. [26] reported that 19.8% still recommended alternating paracetamol and ibuprofen, 38.4% prescribed antipyretics based on specific temperature thresholds rather than child discomfort, and 16.9% would prescribe antipyretics to prevent post-vaccination adverse events. Although antipyretic prescription based on child discomfort increased from 38.2% in 2018 to 61.6% in 2024, this practice remained more frequent among primary care pediatricians than hospital pediatricians (69.5%vs. 56.0%; p = 0.0007). Likewise, Mutic et al. [28] found that only 9.3% of Australian emergency department doctors and nurses adhered to evidence-based recommendations, while 46.8% endorsed combined antipyretic therapy and 36.0% recommended antipyretics to prevent febrile convulsions. Milani et al. [29] also reported persistent misunderstandings among final-year medical students, with only 2.4% considering child discomfort rather than temperature as the criterion for antipyretic use and regional differences in misconceptions regarding brain damage, physical methods, and the beneficial effects of fever. Collectively, these findings suggest that inappropriate fever management may reflect the persistence of culturally and professionally transmitted misconceptions within healthcare systems, beyond caregiver educational level alone.

In the present study, the presence of chronic or congenital conditions was not associated with differences in fever-management adequacy. Although caregivers of children with chronic illnesses might theoretically demonstrate greater familiarity with healthcare systems and symptom monitoring, previous evidence suggests that increased contact with healthcare settings does not necessarily translate into adequate fever-related knowledge or practices. Persistent fever phobia and inappropriate fever-management beliefs have been reported among caregivers in both outpatient and inpatient settings, despite fever-related education during hospitalization, with over 74% of hospitalized caregivers maintaining misconceptions about fever risks even after receiving formal guidance [7]. Accordingly, these findings suggest that inappropriate fever-management practices may remain relatively consistent regardless of previous clinical experience or underlying health conditions.

The results of this study have relevant implications for clinical practice and public health planning. Identifying specific risks and priority groups in home-based fever management provides valuable input for targeted health actions, with the potential to reduce avoidable emergency visits and optimize healthcare resource utilization [11]. The present findings are consistent with those reported by Pitoli et al. (2021) and Gomide et al. (2011) across different Brazilian settings, suggesting that the observed patterns may reflect relatively stable behaviors within the Brazilian population.

This study has some limitations that should be considered when interpreting the findings. As an observational study, it was not designed to establish causal relationships, but rather to identify patterns and associations related to fever management practices among caregivers. Potential selection bias may have occurred because part of the sample was recruited online and participation was voluntary, which could have increased the representation of caregivers with greater digital access or interest in health-related topics. To reduce this limitation, the study adopted a complementary in-person recruitment strategy, broadening access to participants who might not have been reached through digital means. Importantly, comparative analyses showed no significant differences between the online and in-person recruitment modalities [30], supporting the internal consistency of the findings.

The final sample size substantially exceeded the minimum required, which strengthened the precision of the estimates and reduced the likelihood of type II error. Nevertheless, some subgroup analyses should still be interpreted with caution, as they may have had lower statistical power. Because of the cross-sectional design, the association between waiting time and appropriate fever management should not be interpreted causally. One possible explanation is that caregivers with greater previous experience may feel more confident monitoring fever at home before seeking care. Although the analyses considered relevant sociodemographic and behavioral variables, residual confounding related to clinical severity or contextual factors cannot be fully excluded, as is common in real-world observational research.

The use of a composite all-or-none outcome may not capture the full complexity of caregiver decision-making in everyday situations. However, this conservative approach was intentionally adopted to identify participants who simultaneously met all predefined criteria for appropriate fever management, thereby avoiding overestimation of adequate practices. Thus, the reported prevalence should be interpreted as a stringent estimate of complete adherence to recommended management, rather than as a direct measure of clinically unsafe behavior. Finally, although the questionnaire was adapted from previous studies and reviewed to ensure conceptual adequacy, it did not undergo formal psychometric validation in the target population. Even so, the use of standardized items, predefined response categories, and complementary recruitment procedures helped minimize measurement inconsistencies and strengthen the reliability of the collected data.

In summary, childhood fever management remains below recommended standards, particularly with regard to diagnostic accuracy and appropriate antipyretic use, and appears to be primarily influenced by behavioral and perceptual factors. Strengthening evidence-based educational strategies, with emphasis on health literacy and safety-netting principles, may contribute to greater caregiver autonomy, improved home-based fever management, and reduced avoidable emergency care utilization. These findings provide relevant support for public health planning and encourage further investigations addressing social determinants, beliefs, and cultural practices in similar contexts.

## Funding sources

This research did not receive any specific grant from funding agencies in the public, commercial, or not-for-profit sectors.

## Use of generative artificial intelligence

During the preparation of this manuscript, the authors used ChatGPT (OpenAI) as a language-support tool, to assist with language editing and improvement of clarity. After using this tool, the authors reviewed and edited all content and take full responsibility for the final version.

## Data availability

The dataset generated and/or analyzed during the current study is available from the corresponding author upon reasonable request and in accordance with ethical and privacy restrictions.

## Conflicts of interest

The authors declare no conflicts of interest.

## Appendix Supplementary materials

Image, application 1
