# Supplementary material for: Parental management of childhood fever and associated factors in southeastern Brazil: a cross-sectional study
Source: J Pediatr (Rio J). 2026 Jul 28;102(5):101581. doi: 10.1016/j.jped.2026.101581 (PMC13446317; doi:10.1016/j.jped.2026.101581)
Supplement: Supplementary file 1 [file mmc1.docx]

**Parental management of childhood fever and associated factors in southeastern Brazil: a cross-sectional study**

**Short title:** Parental Management of Childhood Fever

This file provides detailed methodological, ethical, and operational information to ensure transparency and reproducibility.

# Supplementary Table 1. STROBE statement for cross-sectional studies.

**Supplementary Table 2**. Distribution of responses for individual components of the composite outcome.

**Supplementary Table 3.** Frequencies of adherence and failure for each component of the composite outcome.

**Supplementary Figure 1.** Forest plot of the only factor independently associated with appropriate pediatric fever management in multivariable logistic regression analysis.

# Supplementary Appendix 1. English-translated version of the questionnaire.

# Supplementary Appendix 2. Detailed comparison between the original fever management questionnaire and the adapted version used in the present study.

# Supplementary Table 1. STROBE statement for cross-sectional studies.

|  | **Item No** | **Recommendation** | **Page No** |
| --- | --- | --- | --- |
| **Title and abstract** | 1 | (*a*) Indicate the study’s design with a commonly used term in the title or the abstract | 1 |
|  |  | (*b*) Provide in the abstract an informative and balanced summary of what was done and what was found | 1 |
| **Introduction** | | | |
| Background/rationale | 2 | Explain the scientific background and rationale for the investigation being reported | 3 |
| Objectives | 3 | State specific objectives, including any prespecified hypotheses | 4 |
| **Methods** | | | |
| Study design | 4 | Present key elements of study design early in the paper | 4 |
| Setting | 5 | Describe the setting, locations, and relevant dates, including periods of recruitment, exposure, follow-up, and data collection | 5 |
| Participants | 6 | (*a*) Give the eligibility criteria, and the sources and methods of selection of participants | 5 |
| Variables | 7 | Clearly define all outcomes, exposures, predictors, potential confounders, and effect modifiers. Give diagnostic criteria, if applicable | 5 |
| Data sources/ measurement | 8 | For each variable of interest, give sources of data and details of methods of assessment (measurement). Describe comparability of assessment methods if there is more than one group | 6 |
| Bias | 9 | Describe any efforts to address potential sources of bias | 4 |
| Study size | 10 | Explain how the study size was arrived at | 4 |
| Quantitative variables | 11 | Explain how quantitative variables were handled in the analyses. If applicable, describe which groupings were chosen and why | 5 |
| Statistical methods | 12 | (*a*) Describe all statistical methods, including those used to control for confounding | 6 |
|  |  | (*b*) Describe any methods used to examine subgroups and interactions | 6 |
|  |  | (*c*) Explain how missing data were addressed | 6 |
|  |  | (*d*) If applicable, describe analytical methods taking account of sampling strategy | 6 |
|  |  | (*e*) Describe any sensitivity analyses | 6 |
| **Results** | | | |
| Participants | 13 | (a) Report numbers of individuals at each stage of study—eg numbers potentially eligible, examined for eligibility, confirmed eligible, included in the study, completing follow-up, and analysed | 6 |
|  |  | (b) Give reasons for non-participation at each stage | - |
|  |  | (c) Consider use of a flow diagram | - |
| Descriptive data | 14 | (a) Give characteristics of study participants (eg demographic, clinical, social) and information on exposures and potential confounders | 7 |
|  |  | (b) Indicate number of participants with missing data for each variable of interest | - |
| Outcome data | 15 | Report numbers of outcome events or summary measures | 7 |
| Main results | 16 | (*a*) Give unadjusted estimates and, if applicable, confounder-adjusted estimates and their precision (eg, 95% confidence interval). Make clear which confounders were adjusted for and why they were included | 7 |
|  |  | (*b*) Report category boundaries when continuous variables were categorized | - |
|  |  | (*c*) If relevant, consider translating estimates of relative risk into absolute risk for a meaningful time period | - |
| Other analyses | 17 | Report other analyses done—eg analyses of subgroups and interactions, and sensitivity analyses | - |
| **Discussion** | | | |
| Key results | 18 | Summarise key results with reference to study objectives | 10 |
| Limitations | 19 | Discuss limitations of the study, taking into account sources of potential bias or imprecision. Discuss both direction and magnitude of any potential bias | 12 |
| Interpretation | 20 | Give a cautious overall interpretation of results considering objectives, limitations, multiplicity of analyses, results from similar studies, and other relevant evidence | 10 |
| Generalisability | 21 | Discuss the generalisability (external validity) of the study results | 12 |
| **Other information** | | | |
| Funding | 22 | Give the source of funding and the role of the funders for the present study and, if applicable, for the original study on which the present article is based | 1 |

**Supplementary Table 2**. Distribution of responses for individual components of the composite outcome.

| **Variable** | **N (%)** |
| --- | --- |
| **Has a thermometer at home** |  |
| Yes | 822 (97.05) |
| No | 25 (2.95) |
| **Type of thermometer used** |  |
| Does not use or does not have a thermometer* | 29 (3.42) |
| At least one digital thermometer** | 765 (90.32) |
| Non-digital thermometer only*** | 53 (6.26) |
| **Knows how to use thermometer properly** |  |
| Yes | 833 (98.35) |
| No | 14 (1.65) |
| **Temperature considered as fever** |  |
| 37.0 - 37.5°C | 72 (8.50) |
| 37.5 - 38.0°C | 592 (69.89) |
| 38.0 - 38.5°C | 138 (16.29) |
| 38.5 - 39.0°C | 39 (4.61) |
| Above 39.0°C | 6 (0.71) |
| **Temperature measurement route** |  |
| Axillary | 807 (95.28) |
| Oral | 28 (3.30) |
| Rectal | 1 (0.12) |
| Tympanic | 11 (1.30) |
| **Use of antipyretics** |  |
| Yes | 816 (96.34) |
| No | 31 (3.66) |
| **Type of antipyretic used** |  |
| Combination therapy**** | 243 (28.70) |
| Dipyrone (alone) | 365 (43.09) |
| Ibuprofen (alone) | 60 (7.08) |
| Does not use antipyretics | 8 (0.94) |
| Others antipyretic regimens | 15 (1.77) |
| Paracetamol (alone) | 156 (18.42) |
| **Parameter used for dose calculation** |  |
| Previous medical prescriptions | 107 (12.64) |
| Child’s age | 52 (6.14) |
| Other | 8 (0.94) |
| Child’s weight | 680 (80.28) |
| **Use of antibiotics** |  |
| Yes | 36 (4.25) |
| No | 811 (95.75) |

* Includes participants who reported not having a thermometer at home and relying on alternative methods such as placing the palm of the hand on the child’s forehead, subjective assessment of chills, borrowed thermometers, or thermometers available in pharmacies or healthcare facilities. ** Includes participants who reported using exclusively digital thermometers or combinations involving digital devices (digital + infrared, digital + mercury-column, or all three types). In Brazil, the commercialization of mercury-containing devices was prohibited by the Brazilian Health Regulatory Agency (ANVISA) through RDC No. 933/2024. *** Includes participants who reported using only infrared thermometers, only mercury-column thermometers, or both. **** Includes participants who reported alternating or combining two or more antipyretics commonly available in Brazil for fever management, particularly dipyrone, paracetamol, and ibuprofen, either concomitantly or sequentially after perceiving insufficient fever reduction with a single agent.

**Supplementary Table 3.** Frequencies of adherence and failure for each component of the composite outcome.

| **Variable** | **Adherence n (%)** | **Failure n (%)** |
| --- | --- | --- |
| Having a thermometer at home | 822 (97.05) | 25 (2.95) |
| Use of at least a digital thermometer | 765 (90.32) | 82 (9.68) |
| Correct thermometer handling | 833 (98.35) | 14 (1.65) |
| Defining fever as temperature ≥ 37.5°C | 592 (69.89) | 255 (30.11) |
| Axillary temperature measurement | 807 (95.28) | 40 (4.72) |
| Use of antipyretics | 816 (96.34) | 31 (3.66) |
| Use of a single recommended antipyretic | 581 (68.59) | 266 (31.40) |
| Weight-based dose calculation | 680 (80.28) | 167 (19.71) |
| Absence of antibiotic use | 811 (95.75) | 36 (4.25) |

Adherence was defined according to the operational criteria established for the composite outcome and based on current recommendations of the Brazilian Pediatric Society (SBP). Adequate fever management practices included: having a thermometer at home; use of at least a digital thermometer; correct thermometer handling; defining fever as a temperature ≥ 37.5°C; axillary temperature measurement; use of antipyretics; use of a single recommended antipyretic (dipyrone, paracetamol, or ibuprofen); weight-based dose calculation; and absence of antibiotic use. Although current pediatric guidelines recommend antipyretic administration primarily according to the child’s discomfort or prostration rather than isolated temperature values, these clinical parameters were not specifically evaluated in the questionnaire. Therefore, reported use of antipyretics was operationally considered an adequate practice for the corresponding individual component, while classification of overall appropriate fever management additionally required fulfillment of the remaining eight composite outcome criteria, including appropriate antipyretic selection and weight-based dosing practices.

**Supplementary Figure 1.** Forest plot of the only factor independently associated with appropriate pediatric fever management in multivariable logistic regression analysis.

OR, *odds ratio;* CI, confidence interval.

# Supplementary Appendix 1. English-translated version of the questionnaire

**QUESTIONNAIRE**

Do you currently live in the municipality of Lavras, Minas Gerais, Brazil?
( ) Yes. ( ) No.

Caregiver’s date of birth: ___.

What is your occupation? ___.

What is your self-declared race? ( ) White.( ) Black.( ) Asian.( ) Brown ( ) Indigenous ( ) Prefer not to answer.

What is your relationship to the child(ren)?( ) Father.( ) Mother.( ) Legal guardian.( ) Other.

Do you currently live with a spouse or partner?( ) No.( ) Yes.( ) Prefer not to answer.

How many children do you have? ___.

What is/are the age(s) of the child(ren) (in completed years)? ___.

What is/are the sex(es) of the child(ren)?( ) Male only.( ) Female only.( ) Both.

Does/do the child(ren) rely exclusively on the Brazilian Unified Health System (SUS)?( ) Yes.( ) No.

Estimated weight of the child(ren): ___.

**CHILD’S PREVIOUS MEDICAL HISTORY QUESTIONNAIRE**

1 – Does/do the child(ren) have any comorbidity(ies) and/or disease(s)?( ) No.( ) Yes. Please specify: ___.

2 – Does/do the child(ren) have any congenital condition(s)?( ) No.( ) Yes. Please specify: ___.

3 – Does/do the child(ren) regularly use any medication or supplement?( ) No.( ) Yes. Please specify: ___.

4 – Has there been any episode of febrile seizure?( ) No.( ) Yes.

How many times did the febrile seizure occur? ___.

**QUESTIONNAIRE ON FEVER MANAGEMENT**

1 – Do you have a thermometer at home?( ) No.( ) Yes.

2 – If not, how do you measure fever? ___.

3 – If yes, what type of thermometer do you have?( ) Digital thermometer.( ) Mercury thermometer.( ) Infrared thermometer.
( ) Digital and mercury thermometers.( ) Digital and infrared thermometers.( ) Mercury and infrared thermometers.
( ) I have all three types.( ) Other(s). Please specify: ___.

4 – Do you know how to use the thermometer?( ) No.( ) Yes.

5 – Which of the following temperatures do you consider to be fever?( ) 37.0 to 37.5°C.( ) Above 37.5°C.

( ) 38.0 to 38.5°C.( ) Above 38.5°C.( ) 38.5 to 39.0°C.( ) Above 39.0°C.

6 – Which route do you use to measure temperature with the thermometer?( ) Oral/mouth.( ) Axillary.( ) Rectal.( ) Tympanic.

7 – If your thermometer is mercury-based, how long do you wait before removing it?( ) 1 to 3 minutes.( ) 3 to 5 minutes.( ) More than 5 minutes.( ) I do not have a mercury thermometer.

8 – If your thermometer is digital, how long do you wait before removing it?( ) 1 to 3 minutes.( ) 3 to 5 minutes.( ) More than 5 minutes.( ) After the sound signal.( ) I do not have a digital thermometer.

9 – If your thermometer is infrared, how long do you wait before removing it?( ) 1 to 3 minutes.( ) 3 to 5 minutes. ( ) More than 5 minutes.( ) After the sound signal.( ) I do not have an infrared thermometer.

10 – How long do you wait before seeking medical care after identifying that your child has a fever?( ) I do not wait and seek medical care immediately.( ) I wait 6 to 12 hours.( ) I wait 12 to 24 hours.( ) I wait 24 to 36 hours.( ) I wait 36 to 48 hours.( ) I wait 48 to 72 hours.

11 – When you identify fever in your child, do you administer antipyretics (medications used to reduce fever)? ( ) Yes.( ) No.

12 – If yes, which antipyretic do you use most frequently?( ) Dipyrone.( ) Paracetamol (Tylenol®).( ) Ibuprofen. ( ) Any of the three.( ) Dipyrone + paracetamol.( ) Dipyrone + ibuprofen.( ) Paracetamol + ibuprofen. ( ) I combine all three (dipyrone + paracetamol + ibuprofen).( ) None, I do not use antipyretics.

( ) Other(s). Please specify: ___.

13 – When administering antipyretics, the dosage used is mainly based on:( ) Child’s weight.( ) Child’s age.

( ) Body surface area.( ) Medical prescription or previous prescriptions.( ) Other. Please specify: ___.

14 – Do you know the adverse effects that may be caused by antipyretics?( ) Yes.( ) No.

15 – If yes, please mention one: ___.

16 – When you identify fever in your child, do you administer antibiotics?( ) Yes.
( ) No.

17 – If yes, which antibiotic is administered?( ) Amoxicillin.( ) Amoxicillin-clavulanate.( ) Cephalexin.( ) I do not use antibiotics.( ) Other. Please specify: ___.

18 – Do you know the adverse effects that may be caused by antibiotics?( ) Yes.( ) No.

19 – To decide which medication should be used to control fever, from whom do you seek information?( ) Pharmacy clerk.( ) Pharmacist.( ) Physician.( ) Family member.( ) Media/information sources.( ) Dentist.( ) Other. Please specify: ___.

20 – Which non-pharmacological methods do you use to reduce fever?( ) I bathe the child.( ) I use cold water compresses. ( ) I use alcohol-soaked compresses or cotton.( ) None.( ) Other. Please specify: ___.

21 – Do you believe fever has any benefit?( ) Yes. Please explain: ___.( ) No. Please explain: ___.

22 – For you, fever is: ( ) A warning sign showing that something is wrong with the child’s body.( ) A sign of infection. ( ) Part of the body’s defense mechanism.( ) All of the above.

23 – Do you consider fever dangerous?( ) Yes. Please explain: ___.( ) No. Please explain: ___.

24 – Which of the following complications can fever cause? ( ) Seizure.( ) Dehydration.( ) Drowsiness.( ) Weakness/lethargy.( ) Brain damage.( ) All of the above.( ) Fever does not cause complications.

25 – Which of the following sources do you seek first for information about fever?( ) Books.( ) Newspapers. ( ) Television.( ) Internet.( ) Scientific articles.( ) Booklets and guides.( ) Other. Please specify: ___.

26 – Apart from media sources, from whom do you mainly seek information?( ) Healthcare professionals.( ) Family members.( ) Friends/neighbors.( ) More experienced people (e.g., elderly individuals or people who already have children).( ) Other. Please specify: ___.

**SOCIOECONOMIC QUESTIONNAIRE**

1 – Marital status:( ) Single.( ) Married.( ) Separated/divorced.( ) Widowed.( ) Other. Please specify: ___.

2 – How many children do you have?( ) One.( ) Two.( ) Three.( ) Four or more.( ) I do not have children.

3 – Who do you currently live with?( ) Parents and/or other relatives.( ) Spouse/partner and/or children.( ) Friends. ( ) Other. Please specify: ___.

4 – How many people live in your household? ( ) One or two.( ) Three or four.( ) Five or six.( ) More than six.

5 – If you do not live with your parents, is your residence close to your children’s maternal or paternal grandparents, or to other relatives (siblings, cousins, etc.)?( ) Yes.( ) No.

6 – Your residence is:( ) Owned.( ) Provided free of charge.( ) Rented.( ) Financed/mortgaged.( ) Other. Please specify: ___.

7 – Monthly family income (sum of the income of all household workers):( ) 1 to 2 minimum wages.( ) 2 to 3 minimum wages. ( ) 3 to 5 minimum wages.( ) 5 to 10 minimum wages.( ) 10 to 20 minimum wages.( ) More than 20 minimum wages. ( ) No fixed monthly income.

8 – Educational level:( ) No formal education.( ) Elementary school: 1st to 4th grade.( ) Elementary school: 5th to 8th grade.( ) High school.( ) Higher education.( ) Postgraduate education.

# Supplementary Appendix 2. Detailed comparison between the original fever management questionnaire and the adapted version used in the present study.

| **Modifications related to participant characteristics** | | | |
| --- | --- | --- | --- |
| **Original questionnaire item/domain** | **Adaptation/modification performed** | **Type of modification** | **Rationale** |
| Original questionnaire developed for face-to-face application | Adapted for anonymous self-administered online completion | Methodological adaptation | To facilitate remote data collection and improve participant privacy |
| Participant name | Removed from adapted questionnaire | Removal | To preserve anonymity and confidentiality |
| Address, city, state, telephone number, and e-mail | Removed from adapted questionnaire | Removal | To avoid collection of personally identifiable information |
| No geographic eligibility question | Added: “Do you currently live in the municipality of Lavras, Minas Gerais, Brazil?” | Added question | To restrict inclusion to the target municipality and ensure geographic eligibility according to the study design |
| Relationship to the child (father/mother/legal guardian) | Added “Other” option | Response expansion | To improve inclusiveness of caregiver profiles |
| No caregiver occupation question | Added caregiver occupation question | Added question | To improve sociodemographic characterization |
| No race/ethnicity question | Added self-declared race question | Added question | To allow epidemiological characterization |
| No cohabitation/marital structure question | Added question regarding living with spouse/partner, including “Prefer not to answer” option | Added question | To improve family structure assessment while preserving participant autonomy |
| No race/ethnicity question | Added IBGE-based self-declared race categories and “Prefer not to answer” option | Added question | To allow epidemiological characterization and improve standardization according to Brazilian demographic classifications |
| Child age assessed in singular form (“age of the child”) | Expanded to “age(s) of the child(ren) in completed years” | Structural adaptation | To accommodate caregivers responsible for multiple children |
| Child profile included child age only | Added child sex information | Added question | To improve pediatric characterization |
| Child profile included child age only | Added estimated child weight | Added question | To allow interpretation of antipyretic dosing practices and medication administration behaviors |
| Relationship to the child included only “father,” “mother,” and “guardian” | Added “Other” caregiver category | Response expansion | To capture non-traditional caregiving arrangements |
| No healthcare access question | Added question regarding exclusive use of the Brazilian Unified Health System (SUS) | Added question | To evaluate healthcare access profile |
| Number of children question present only as categorical variable | Added open-ended numerical question regarding number of children | Structural adaptation | To allow quantitative analyses |
| Original questionnaire entirely in Portuguese | Entire questionnaire translated and linguistically standardized into English | Linguistic adaptation | To allow international publication and improve readability |
| **Modifications related to child previous medical history** | | | |
| **Original questionnaire item/domain** | **Adaptation/modification performed** | **Type of modification** | **Rationale** |
| No previous medical history section | Added “Child’s Previous Medical History Questionnaire” | New section added | To improve clinical characterization |
| No comorbidity assessment | Added comorbidity/disease question | Added question | To identify chronic clinical conditions |
| No congenital disease assessment | Added congenital condition question | Added question | To characterize pediatric baseline health status |
| No chronic medication/supplement assessment | Added regular medication and supplement use question | Added question | To assess chronic treatment exposure |
| No febrile seizure assessment | Added history of febrile seizure question | Added question | To evaluate prior fever-related complications, given that febrile seizures are among the most common caregiver concerns reported in the literature |
| No question regarding number of febrile seizures | Added “How many times did the febrile seizure occur?” | Added question | To quantify previous febrile seizure burden |
| **Modifications related to fever management** | | | |
| **Original questionnaire item/domain** | **Adaptation/modification performed** | **Type of modification** | **Rationale** |
| Thermometer types limited to digital and mercury thermometers | Added infrared thermometer option | Response expansion | To reflect contemporary thermometer technologies and current household availability |
| Single thermometer selection only | Added combined thermometer alternatives (digital + mercury; digital + infrared; mercury + infrared; all three) | Response expansion | To better represent real-world household thermometer availability |
| Thermometer handling questions lacked explicit opt-out logic | Added thermometer-specific opt-out responses for each device type | Structural adaptation | To improve internal consistency and reduce forced responses |
| Thermometer question lacked open-ended option | Added “Other(s). Please specify” | Response expansion | To capture additional thermometer types |
| Questions regarding thermometer handling time partially merged in the original questionnaire | Separated into independent items for mercury, digital, and infrared thermometers | Structural adaptation | To improve logical consistency and reduce ambiguity |
| Questions only for mercury and digital thermometer handling time | Added specific infrared thermometer timing question | Added question | To improve assessment of contemporary fever measurement practices |
| Digital thermometer response (“after the sound signal”) embedded within mercury thermometer item in the original questionnaire | Created independent response structure for each thermometer type | Structural restructuring | To improve clarity and response validity |
| Mercury thermometer question lacked “I do not have this thermometer” option | Added “I do not have a mercury thermometer” | Response modification | To reduce ambiguity |
| Digital thermometer question lacked “I do not have this thermometer” option | Added “I do not have a digital thermometer” | Response modification | To reduce ambiguity |
| No infrared thermometer timing responses | Added complete infrared timing response structure | Added question | To standardize assessment across thermometer types |
| Temperature measurement route described as “Oral” only | Changed to “Oral/mouth” | Linguistic adaptation | To improve comprehension among respondents with different literacy levels |
| Antipyretic administration question lacked explanatory wording | Added clarification: “(medications used to reduce fever)” | Linguistic adaptation | To improve understanding across different educational levels |
| Antipyretic options limited to isolated drugs | Added combined antipyretic regimens | Response expansion | To assess alternating and combined antipyretic administration practices commonly described among caregivers |
| No option for combined dipyrone + paracetamol | Added combined dipyrone + paracetamol option | Response expansion | To identify simultaneous drug use |
| No option for combined dipyrone + ibuprofen | Added combined dipyrone + ibuprofen option | Response expansion | To identify simultaneous drug use |
| No option for combined paracetamol + ibuprofen | Added combined paracetamol + ibuprofen option | Response expansion | To identify simultaneous drug use |
| No option for all three combined | Added “I combine all three” option | Response expansion | To evaluate potentially inappropriate practices |
| No explicit “none” option in antipyretic combinations | Added “None, I do not use antipyretics” | Response modification | To improve response specificity |
| Antipyretic dosage criteria included “all of the above” and “none of the above” | Simplified answer structure and added open-ended “Other. Please specify” option | Response restructuring | To reduce ambiguity, improve interpretability, and facilitate statistical analysis |
| Antibiotic use structure maintained | Added explicit “I do not use antibiotics” option | Response modification | To improve specificity |
| Medication information sources limited to pharmacy clerk and pharmacist | Added physician, dentist, family member, and media/information sources | Response expansion | To better characterize real-world healthcare-seeking behavior and information pathways |
| Medication information sources included “none of the above” | Replaced with expanded and clinically relevant information-source categories | Response restructuring | To improve ecological validity of healthcare-seeking behavior assessment |
| Medication information sources lacked open-ended alternatives | Added “Other. Please specify” | Response expansion | To increase response flexibility |
| Non-pharmacological methods included “I do not use any of these” | Simplified to “None” | Linguistic adaptation | To improve readability and response clarity |
| Non-pharmacological methods lacked open-ended alternatives | Added “Other. Please specify” | Response expansion | To capture additional fever management practices |
| Fever information sources limited to books, newspapers, television, and internet | Added scientific articles and educational booklets/guides | Response expansion | To reflect contemporary educational and digital information sources |
| Fever information source question evaluated general use only | Modified wording to assess which source respondents seek first | Structural adaptation | To introduce hierarchical evaluation of information-seeking behavior |
| Interpersonal information source question lacked prioritization wording | Added the qualifier “mainly/primarily” | Linguistic adaptation | To improve interpretation of preferred information sources |
| Interpersonal information source question underwent grammatical standardization | Minor wording revision | Linguistic adaptation | To improve readability and consistency |
| Interpersonal fever information sources lacked open-ended alternatives | Added “Other. Please specify” | Response expansion | To capture additional interpersonal sources |
| **Modifications related to socioeconomic characteristics** | | | |
| **Original questionnaire item/domain** | **Adaptation/modification performed** | **Type of modification** | **Rationale** |
| Marital status question lacked open-ended specification field | Added “Other. Please specify” | Response expansion | To improve inclusiveness of relationship categories |
| Number of children question lacked non-parent caregiver option | Added “I have no children” | Response expansion | To accommodate non-parental caregivers |
| Household composition question lacked open-ended specification field | Added “Other. Please specify” | Response expansion | To improve flexibility of household characterization |
| Housing conditions included owned, rented, ceded, and others | Added financed/mortgaged housing option | Response expansion | To improve socioeconomic stratification |
| Family income categories ended at “10–20 minimum wages” | Added “More than 20 minimum wages” category | Response expansion | To improve socioeconomic stratification of higher-income households |

The adaptations implemented in the present study were designed to preserve the conceptual structure of the original questionnaire while improving contextual applicability, epidemiological characterization, response specificity, and suitability for anonymous online administration. Importantly, the modifications did not alter the core constructs assessed by the original instrument regarding caregiver fever management practices, thereby maintaining conceptual comparability with prior studies while improving methodological clarity and contemporary relevance.
